# Validity of New Technologies That Measure Bone-Related Dietary and Physical Activity Risk Factors in Adolescents and Young Adults: A Scoping Review

**DOI:** 10.3390/ijerph18115688

**Published:** 2021-05-26

**Authors:** Alyse Davies, Yumeng Shi, Adrian Bauman, Margaret Allman-Farinelli

**Affiliations:** 1 Charles Perkins Centre, Nutrition and Dietetics Group, School of Life and Environmental Sciences, The University of Sydney, Sydney, NSW 2006, Australia; yumeng.shi@sydney.edu.au (Y.S.); margaret.allman-farinelli@sydney.edu.au (M.A.-F.); 2 Prevention Research Centre, School of Public Health, The University of Sydney, Sydney, NSW 2006, Australia; adrian.bauman@sydney.edu.au

**Keywords:** adolescents, bone, diet, nutrition, physical activity, technologies, validity, young adult

## Abstract

New technologies may improve the validity of dietary and physical activity assessment and thereby associated findings for lifestyle-related bone health research. This scoping review mapped the evidence for the validity of new technologies that measure bone-related dietary and physical activity risk factors in adolescents and young adults. A systematic literature search was conducted using seven electronic databases for peer-reviewed studies published from January 2008 to 2021. Four studies from four countries were deemed eligible and included in the qualitative synthesis for this review. Two studies assessed diet, reporting the validity or usability of apps. Apps were shown to be a valid tool to measure the dietary intake of vitamin D (r = 0.84) and calcium (r = 0.63). Two studies assessed physical activity and reported the validity of wearable devices to measure impact loading. Hip-worn raw acceleration output correlated positively with ground reaction forces (GRF) for both studies (r range = 0.50–0.87), but wrist-worn accelerations and loading outcomes differed between studies, reporting poor to strong correlations (r range = 0.17–0.87). More research to provide robust evidence concerning validity, reliability, usability and engagement for the use of newer technologies is needed for future diet and physical activity bone research.

## 1. Introduction

Diet and physical activity are important modifiable lifestyle factors to achieve maximal bone health and peak bone mass (PBM) [1]. Peak bone mass is described as the maximal amount of bone mass achieved during growth and development, before accrual ceases or plateaus, which can occur in the late teenage years or in young adulthood [2]. The United States National Osteoporosis Foundation conducted a systematic review of the literature on PBM development and lifestyle factors in 2016 [3]. They applied an evidence grading system describing the strength of available evidence for individual modifiable factors that are either beneficial or detrimental for PBM achievement. Grade A evidence included positive effects of calcium intake, physical activity and exercise. Grade B evidence was assigned for the benefit of vitamin D and dairy foods on bone. Grade C evidence included positive findings for protein, fiber, fruits and vegetables and the detrimental effects of cola and caffeinated beverages on bone.

There has been exponential growth in the use of technology globally. Most adults in advanced economies have access to the internet with the highest rates reported in South Korea (94%), Australia (93%) and Canada (90%) with rates over 80% for the USA, UK, Spain, Israel and Germany [4]. Further, 76% of adults own a smartphone in advanced economies [5]. Commercially available wearable technologies to self-monitor health are increasing, with the global market net worth estimated at USD 87 billion by 2023 [6]. As the technological landscape has advanced, so has the development of new tools to measure diet and physical activity [7]. Such advances have the potential to improve health behaviors specifically related to bone health in adolescents and young adults in free-living settings during the period of PBM attainment.

The use of technology for dietary intake assessment has expanded rapidly over the past decade. The public has indicated its willingness to share diet and physical activity collected [8]. A review of the literature up to 2013 evaluated the feasibility and validity of mobile phones to assess dietary intake, showing similar validity when compared to conventional methods [9], and newer reviews document the increasing use of digital imaging methods being validated [10,11]. Some examples in young adults include the relative validity of the electronic Dietary Intake Assessment (e-DIA) app, and 24-h recalls reported moderate to strong correlations and no differences between methods for mean energy, nutrients (included calcium) or food groups (included fruit, vegetables and dairy products) [12,13]. The Eat and Track (EaT) app measured eating out habits and reported acceptable agreement for most nutrient densities at a group level when compared to the 24-h recall method [14]. The mobile Food Record image-based dietary assessment method for mobile devices has demonstrated validity with doubly labeled water in a community sample that included young adults [15]. While new technologies for dietary assessment have been developed and validated, less research has investigated the validity of technologies for assessing dietary intake with the aim of improving bone-related outcomes.

Technologies, particularly accelerometry, have expanded in physical activity research. While these technologies have been validated [16,17], the outcomes are often time spent in different activity intensities which are calculated using accelerometer cut points validated against energy expenditure (EE). While this is appropriate when examining other health outcomes, it is not an appropriate accelerometer measure for bone health as counts or metabolic equivalents (METs) do not directly provide information on impact loading of bone.

Loading forces in the gravitational plane contribute to bone mass [18]. Peak impact load is described as the greatest force during an initial landing, while the loading rate is the velocity of the application of force [19]. The average resultant force is the same acceleration from the result of several forces. Loads from the impact with the ground are described as ground reaction forces (GRF) [18]. New generation accelerometer models (i.e., Actigraph and GENEActive models) that allow raw signal detection are reasonably accurate when measuring GRF applied to the skeleton [20], while other wearables including fitness trackers, some smart phone apps and step counters would be inappropriate as they fail to measure forces as a vector. These new generation models can assess body acceleration in raw acceleration values within a dynamic operating range (±6–8 g) with a sampling frequency up to 100 Hz, which has shown to be important for capturing short and high-impact accelerations beneficial to bone. Counts are usually processed by a user-defined epoch or period of time (mostly between 1 and 60 s), with shorter epochs of 1 s more accurately capturing intermittent physical activity at any intensity [20,21]. Force plates are considered the “gold standard” for GRF measurement and accelerometer calibration [22]. As laboratory-based methods such as force plates cannot be used in free-living or community-based studies, it is important that devices such as accelerometers are shown to be a reasonably accurate surrogate measure of GRF for bone-related research.

To understand the extent, range and nature of research using new technologies for bone-related lifestyle factors, a scoping study was selected. Scoping reviews are useful to identify knowledge gaps and the types of available evidence in the field [23,24]. There is strong evidence suggesting that calcium and vitamin D are important nutrients for maximization of bone mineral density (BMD) and strength during periods of growth and development [3]. Similarly, impact loading physical activity (i.e., movements that produce high load magnitudes such as jumping) have positive effects on bone geometry, strength and mass [3,25]. Given modifiable risk factors for bone have been widely studied, it is important that new technologies that measure such risk factors produce reasonably accurate data. This scoping review maps the evidence relating to the validity of the technologies that measure bone-related dietary and physical activity risk factors in healthy adolescents and young adults. Secondary outcomes include the reliability and usability of these technologies.

## 2. Materials and Methods

The conduct of this scoping review followed the updated Joanna Briggs Institute guidance for scoping reviews [26]. The protocol was developed and published on the Open Science platform https://osf.io/8uhg5/.

### 2.1. Inclusion Criteria

#### 2.1.1. Participants

Studies were included if the participants were aged between 13 and 35 years, free from disease or illness. If other age groups were reported in addition to the age group of interest, articles were excluded if data could not be extracted separately for the age of interest. Those pregnant or breastfeeding were excluded.

#### 2.1.2. Concept

Studies investigating the validity of new technologies that measure bone-related dietary and/or physical activity risk factors in adolescents and young adults were included. Secondary outcomes related to the feasibility or usability of the new technologies. New technologies included online web-based tools (i.e., web-based 24-h dietary recalls or food frequency questionnaires); mobile-based tools or apps; or wearable devices (i.e., body-worn monitors including cameras or accelerometers).

#### 2.1.3. Context

This review considered studies conducted in the community at large. This includes any free-living population such as schools, universities, workplaces or the home. Hospital inpatient settings were excluded due to the associated diseases or illness that may impact dietary intake or physical activity levels.

### 2.2. Types of Studies

Only peer-reviewed primary studies were selected for review if they fulfilled the following criteria: quantitative study designs, written in English, analyzes/discusses the validity, reliability or usability of new technologies that measure bone-related dietary and/or physical activity risk factors. The time frame was set from 2008 (debut of smartphone apps) to January 2021. Studies that solely reported the validity of the new technologies without reference to bone health were excluded. Conference abstracts were also excluded.

### 2.3. Search Strategy

An initial limited search of MEDLINE and Scopus was undertaken to identify articles on the topic. A full search strategy was developed based on keywords contained in the titles and abstracts of applicable articles, and the index terms used to classify the articles with assistance from a research librarian. A full search was conducted in seven electronic databases, including Ovid (MEDLINE, EMBASE and Cochrane Central Register of Controlled Trials (CENTRAL)), CINAHL, Scopus, Web of Science and Compendex. A search was also conducted in the Cochrane Database of Systematic Reviews via Ovid for reference lists of reviews. The search strategy for MEDLINE is shown in Supplementary Table S1, conducted in January 2021. A manual review of main authors and a hand search of the reference lists of relevant articles (i.e., randomized controlled trials) for the validation study of the assessment tool were conducted. Reference lists of articles selected for full-text review were screened for additional papers. Clinical trial registers for trials underway or completed were searched, and information was requested if the trial was applicable to the scoping review question.

### 2.4. Selection Process

**Figure 1:**
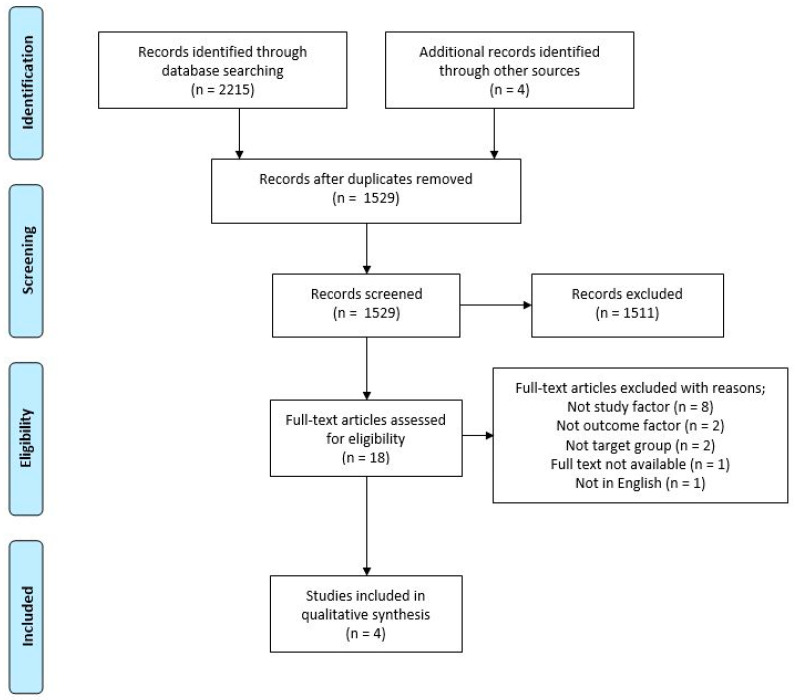
Flowchart of identification and screening process for selection of studies relating to the validity of new technologies that measure bone-related dietary and physical activity risk factors in healthy adolescents and young adults.

The identified records from the full search were imported to EndNote X9.2 (Clarivate Analytics, Philadelphia, PA, USA) for screening. Decisions in each step were recorded in EndNote. Titles and abstracts were screened against the inclusion criteria by two reviewers (A.D. and Y.S.) independently. For the studies with the potential to be included, full-text articles were retrieved and attached in EndNote. Two reviewers screened them against the inclusion and exclusion criteria independently. Reasons for exclusion at this stage were recorded and reported in a PRISMA diagram (Figure 1), and a list of excluded studies is provided in Supplementary Table S2. Disagreements between two reviewers were solved through discussion between each other or consultation with a third reviewer (M.A.-F. for the dietary component or A.B. for the physical activity component).

### 2.5. Data Charting

The data were charted onto a data charting form based on an existing framework for conducting scoping reviews [23]. Two reviewers independently (A.D. and Y.S.) charted the data. Authors of papers were contacted if missing or additional data needed to be requested. For each included study, the following information was extracted: author(s), year of publication, study location; study population (age, gender and sample size); aims/purpose; methodology; outcome measures (validity, reliability or usability); important results; conclusions and funding source.

### 2.6. Synthesis of Results

The charted results are presented in two tables. The first table includes the study characteristics, and the second table is a summary of the methods and key results of the included studies. A narrative summary describes the charted results. The findings of this review will be used to inform lifestyle interventions targeting bone in adolescents and young adulthood regarding the use of valid, reliable and usable technologies.

## 3. Results

### 3.1. Search Results

A total of 2215 potentially relevant studies were identified using the search strategy on seven electronic databases (Medline = 753, EMBASE = 299, CENTRAL= 74, CINAHL= 433, Scopus = 311, Web of Science = 254 and Compendex = 27) and the Cochrane Database of Systematic Reviews (=64) for reference lists of reviews. Four additional articles were retrieved from reference list searching. This was reduced to 1529 after removal of duplicates. After abstract and title screening, 1511 studies were excluded, leaving 18 full-text articles. Of the 18 studies that were screened in full-text publications for eligibility, only 4 studies were deemed eligible and included in the qualitative synthesis. The flowchart in Figure 1 displays the process of selection. Those full-text studies which were deemed ineligible with reasons are reported in Supplementary Table S2.

### 3.2. Study Characteristics

Table 1 shows the study characteristics. One study was conducted in Canada [27], one from Australia [28], one from the UK [29] and one from the USA [30]. The earliest publication date was 2012 and the most recent was 2020. Two studies assessed physical activity [29,30] and two assessed diet [27,28]. Of the dietary studies, one study examined calcium [28], while the other examined calcium and vitamin D [27]. The sample size ranged from 10 to 50 participants, with *n* = 130 individuals included in this review, 65% of the total being female. One study consisted of only female participants [28], while the others comprised of individuals of both genders equally [27,29,30].

### 3.3. Dietary Measures

The two dietary studies used mobile technology. One reported on the validity and reproducibility comparing the vitamin D calculator app (VDC-app) with 24-h dietary recalls assessing the difference (paired-sample *t*-tests), agreement (Bland–Altman plot and Pearson correlation) and reproducibility (intra-class correlations (ICC) and Wilcoxon signed-rank tests) [27]. The other reported on the usability of the Calci-app using a five-item usability questionnaire [28].

### 3.4. Physical Activity Measures

The two physical activity studies used wearable devices. One study used three tri-axial accelerometers (GT1M, GT3X+ and GENEA) [29], while the other study used one tri-axial accelerometer, the GT9X Link [30]. The positioning of the accelerometers varied from the right hip [29,30], right wrist [29,30] and left wrist [29] to the right ankle [30]. All studies used the sampling rate of 100 Hz for the ActiGraph devices (GT3X+ and GT9X Link), while 80 Hz was used for the GENEA. The GT1M was set to collect data in counts (vertical axis, 1 s epoch). The operating range varied between studies, from 6 [29] to 8 g [30]. The force plate, used to collect GRF, ranged from 960 [29] to 1000 Hz [30]. Both studies [29,30] reported validity comparing the correlations between accelerometer output variables and GRF using force plates, while one study used linear regression analyses to examine the relationship between estimated loading outcomes and BMD [30].

### 3.5. Outcomes

A summary of the validity, reliability and usability of technologies for the measurement of diet and physical activity is presented in Table 2.

#### 3.5.1. Validity and Usability of Mobile Apps for Diet and Bone Health

One study [27] reported on the validity and reproducibility of an app. Results from several different analyses suggest the app to be a valid dietary assessment tool for the intake of vitamin D and calcium. Three day mean vitamin D and calcium intakes were positively correlated (r range = 0.63–0.98) and were not significantly different between measures (*p* > 0.05). Reliability analyses were less consistent with a significant difference (*p* = 0.002) between reporting days 1 vs 3 for vitamin D using ICC, but there was no difference (*p* > 0.05) using Wilcoxon signed-rank. One study [28] reported on the usability of an app. In total, 83% of the total participants who completed the study also completed the usability questionnaire. Most participants found the app convenient and easy to use (61%), but time-consuming (42%).

#### 3.5.2. Validity of Accelerometers for Physical Activity Assessment to Estimate Impact Loading

One physical activity study [29] used the GT1M accelerometer. Counts were positively correlated with peak impact force (r = 0.85, *p* < 0.05), average resultant force (r = 0.73, *p* < 0.05) and peak loading rate (r = 0.76, *p* < 0.05).

Both studies [29,30] presented correlations for combined activities for the hip- and wrist-worn accelerometers. Hip vertical accelerations correlated with average resultant (r = 0.85, *p* < 0.05; r = 0.82, *p* < 0.05) and peak loading rate (r = 0.76, *p* < 0.05; r = 0.70, *p* < 0.05) for the GT3X+ and the GENEA, respectively [29]. For the GT9X, hip vertical accelerations correlated with both vertical GRF and loading rate (r = 0.50–0.57, *p* < 0.001) [30].

Hip resultant accelerations correlated with average resultant (r = 0.87, *p* < 0.05; r = 0.85, *p* < 0.05) and peak loading rate (r = 0.70, *p* < 0.05; r = 0.63, *p* < 0.05) for the GT3X+ and GENEA, respectively [29]. For the GT9X, hip resultant accelerations correlated with resultant GRF (r = 0.69, *p* < 0.001) and resultant loading rate (r = 0.74, *p* < 0.001) [30].

Wrist resultant accelerations correlated with average resultant (r = 0.82, *p* < 0.05; r = 0.87, *p* < 0.05) and loading rate (r = 0.79, *p* < 0.05, r = 0.81, *p* < 0.05) for the GT3X+ and GENEA, respectively [29]. For the GT9X, wrist resultant accelerations correlated significantly with resultant loading rate (r = 0.17, *p* < 0.001), but not resultant GRF (r = 0.01, *p* = 0.815) [30].

## 4. Discussion

This scoping review is the first to map the evidence relating to the validity of new technologies that measure bone-related dietary and physical activity risk factors in healthy adolescents and young adults. Among four studies identified, three studies assessed validity and one assessed usability. Apps were shown to be a valid tool to measure dietary vitamin D and calcium intake and most participants found apps convenient and easy to use. Wearable devices were demonstrated to be reasonably accurate for measurement of impact loading previously demonstrated to be beneficial for bone, although not specifically measured in this study. No validation studies for web-based tools were identified. As adolescence and young adulthood are critical periods of bone development and PBM achievement, more uptake and testing of new technologies in this population are required to expand and confirm the evidence given only a small number of studies were identified.

While digital technologies for the assessment of dietary intake are becoming more widespread [10], there has been a lack of adoption of these technologies by bone researchers. Bone researchers would benefit from using newer dietary assessment technologies as this would make monitoring diet easier. Compared to conventional dietary assessment methods such as pen and paper food records and questionnaires, digital technologies have overcome many methodological limitations. Digital technologies (including online web-based or mobile-based tools) collect data in real time, thereby limiting recall bias and participant burden as entries can be completed faster [31]. Additional features including saving favorite foods, scanning barcodes and the ability to send reminders to log data increase the accuracy of the data collected. As coding and nutrient analyses are automated, the time required to access data output is reduced, saving on cost and researcher burden. Another method which bone researchers could explore is digital imaging [32,33]. The participants take photographs of their food selection and plate waste and send the images to the researchers’ server for analysis. A validation study using this method reported promising results for calcium [34]. If bone researchers use new technologies, this may benefit dietary interventions on two fronts. Firstly, when conducting randomized controlled trials, the collection of dietary information would be easier given the additional features and greater acceptability of these methods [10]. Secondly, if the population at large is trying to improve its bone health, apps could be used for self-monitoring purposes, for example, if diagnosed with osteopenia or osteoporosis, the use of apps may help to increase awareness and monitor the intake of foods and/or nutrients beneficial or detrimental to bone health.

Vitamin D and calcium are two important nutrients for bone health [1], yet this review located only one study validating an app for assessing the intake of these nutrients to prevent bone diseases [27]. Although the VDC-app was shown to be a valid tool for measuring the intake of vitamin D and calcium, no conclusions can be made based on the limited studies identified. Although recent reviews have documented the validity of mobile-based tools or apps [10,11,35], very few studies validate nutrients, foods or food groups beneficial to bone. Only a handful of studies report the relative validity of apps for assessing calcium or calcium densities in adolescents or young adults [12,36,37,38], while others exclude calcium [14,39,40]. None, however, report the validation of dietary vitamin D. Food groups including fruit, vegetables and dairy products have beneficial effects on bone, yet the relative validity of apps to measure the intake of food groups has only been reported by one study [13]. There are a range of lifestyle apps available on the App Store or Google Play, with MyFitness Pal (MyFitnessPal Inc, San Francisco, CA, USA) being the most popular among young adults [8]. Other available apps specifically track fruit and/or vegetables, calcium or caffeine, but investigations of the validity of dietary assessment within these apps are required and may be more burdensome than an image-based method.

There are a range of wearable devices that can be used for the assessment of diet or physical activity [7]. As wearables are an emerging field for dietary assessment (i.e., sensors), it was not surprising that this review found no validation studies. Device-based physical activity assessment tools such as general-use wearables, heart rate monitors, global positioning sensors (GPS), pedometers and accelerometers have been widely used and validated [7]. Although accelerometers are frequently used to measure physical activity, studies using accelerometers to examine physical loading are limited. Raw acceleration data can be used to measure impact loading, but calibration with mechanical measures relevant to bone health is required, i.e., GRF using force plates. A study conducted in children found new generation accelerometer models to be reasonably accurate to measure impact loading, although they were shown to overestimate GRF [20]. This scoping review identified two studies that aimed to validate acceleration data with GRF in adolescents or young adults, with one also conducting linear regression analyses to examine the relationship between estimated loading outcomes and BMD. Accelerometer counts and raw acceleration output correlated positively with GRF, which suggests that accelerometry may be reasonably accurate as a proxy measure to assess impact loading beneficial for bone. Peak accelerations during walking, running, jumping and combined activities were positively correlated with GRF output variables [29,30]. Further, a linear regression using GRF and loading rates and/or accelerations showed significant associations with total body and hip BMD [30].

The response of bone to mechanical stimuli is threshold driven [25]. A study examining the effects of a vertical jumping exercise program (50 vertical jumps with a mean height of 8.5 cm, 6 days a week) produced mean ground reactions of three times the body weight (BW) in pre-menopausal women and significant increases in femoral BMD [41]. A later study conducted on pre-menopausal women reported cut points associated with the loading rate of 43 BW per s^−1^ to be beneficial to bone as it led to increases in BMD at the trochanter and femoral neck; however, these cut points were specific to the GENEActiv and Actigraph GT3X+ models [42]. Research on post-menopausal women has suggested that acceleration levels above 3.9 g seen during impact exercises have shown to increase BMD in the proximal femur [43]. The custom accelerometer used to establish this threshold had an operating range of ±16 g and a sampling rate of 400 Hz which is not comparable to the system characteristics of the studies reported in this scoping review.

It is important to be cautious when comparing studies and interpreting findings with different accelerometers and system characteristics (i.e., system operating range and sampling rates). Operating ranges and sampling rates can influence measures of peak impact loading with the signal frequency sensitive to the sampling rate and the signal magnitude sensitive to the operation range [44]. In this review, one study used three accelerometers, where the operating range was set at 6 g and the sampling rates differed between each device: GT1M (1 s epoch), GT3X+ (100 Hz) and GENEA (80 Hz). The other study used a GT9X Link accelerometer with an operating range of ±8 g and sampling rate of 100 Hz. Previous research has shown that there is a large variation in system characteristics reported in the literature with operating ranges from ±6 to ±40 g and sampling rates from 40 to 2000 Hz [44]. Standardized protocols or raw data on force vectors might be necessary to achieve comparability across models.

Accelerometers can be placed on a range of wear site locations, with the most common placement being the hip or wrist. While previous studies predominantly used hip-worn accelerometers, more recent studies used wrist-worn accelerometers due to higher compliance [45,46]. Both studies in this review compared hip- and wrist-worn accelerometers to loading outcomes, but findings differed between studies for wrist-worn accelerometers. Similar to a study conducted on pre-menopausal women [42], one study found wrist-worn monitors to be a viable option for future studies for bone health as moderate to strong correlations with GRF were reported (r range = 0.58–0.87), whereas no significant associations were reported with wrist-based outcomes during any activity (r range = 0.01–0.17) for another study. Overall, wrist-worn accelerometers seem to be less accurate than hip-worn accelerometers for assessing forces relevant to bone health. Significant associations with BMD at the hip and total body suggest that hip-worn accelerometers should be used in bone health studies especially focusing on the lower limbs [30]. Further uptake and validation of objective physical activity measures in free-living settings are important to bone research and future development of bone-specific physical activity guidelines.

A strength of this review is that the protocol for the methodological framework for the scoping study was determined a priori and published on the Open Science platform. Additionally, the review explored the use of new technologies for two modifiable risk factors: diet and physical activity. The main limitation is that no conclusions could be drawn due to the few studies identified; however, this scoping review identified a research gap for future bone research. There were considerable methodological differences which made comparisons across studies challenging. Studies estimating GRF by developing generalized regression equations were excluded even though acceleration data were used in developing the equations. Validation studies for both diet and physical activity may have been missed if bone was not reported as a study factor. As this review focused on the use of technologies to assess already recognized lifestyle risk factors for bone disease, the validation studies did not report a change in bone outcomes but were rather focused on validating the technology.

## 5. Conclusions

While new technologies show potential for the measurement of bone-related dietary and physical activity modifiable risk factors, there was a paucity of studies. More studies are needed to provide robust evidence of validity, reliability and usability for the uptake of these technologies in future diet and physical activity bone research. The use of valid measures that relate to bone mineral deposition and measurement tools that can be used in free-living settings is important for bone-related lifestyle interventions in adolescents and young adults and for the future development of bone-specific physical activity guidelines. It is hoped this review stimulates further research on assessments relevant to bone health outcomes given the direct economic costs associated with osteoporosis, osteopenia and fracture treatment.

## Figures and Tables

**Table 1:** Study characteristics of the included studies (*n* = 4).

Author, Year, Country	Aims/Purpose	Lifestyle Factor/s	Sample Size (*n*)	Age in Years Range, Mean (SD) ^1^	Sex, Female (%)	Funding
Goodman [27] 2015 Canada	To establish the validity and reproducibility of the dietary component of a mobile vitamin D calculator app	Vitamin D Calcium	Vitamin D (50) Calcium (48)	18–25 22.0 (2.0)	50	Canadian Institutes for Health Research Frederick Banting Doctoral Scholarship (grant #596230)
Tay [28] 2017 Australia	To assess the usability and acceptability of Calci-app in young women to self-monitor dietary calcium intake and its potential for use in a bone health mHealth behavior change intervention	Calcium	40	18–25 N/R ^2^	100	Nowpos M-Solutions Pvt Ltd. (Hyderabad, India) for supporting the development of Calci-app; no conflicts of interest to declare.
Rowlands [29] 2012 UK	To assess the relationship of accelerometer output, in counts (ActiGraph GT1M) and as raw accelerations (ActiGraph GT3X+ and GENEA), with GRF ^3^ in adults	Physical Activity	10	N/R^2^ Males; 26.4 (4.0) Female; 32.4 (10.5) Overall; 29.4 (8.2)	50	No external funding was received for this research. None of the authors have a conflict of interest with ActivInsights or ActiGraph, the manufacturers of the technologies on which this article is based.
Higgins [30] 2020 USA	(1) To assess the concurrent validity of raw accelerometer outputs with GRF ^3^ and loading rates calculated from force plate across a range of simulated habitual physical activities. (2) To identify the optimal wear site among the ankle, hip and wrist with the strongest relationships between accelerometer and force plate and/or skeletal outcomes.	Physical Activity	30	18–35 23.0 (4.5)	50	Faculty Research and Development funds from Elon University. The authors declare no conflict of interest.

^1^ SD = standard deviation; ^2^ N/R = not reported; ^3^ Ground reaction force = GRF.

**Table 2:** Summary of the methods and results of the included studies (*n* = 4).

Ref	Technology Type	Study Type	Outcome Measures	Important Results
[27]	Mobile app Intake of vitamin D and calcium containing foods, beverages and supplements in app. Immediate nutrient feedback is provided relative to recommendations (3 days over 1 month; 2 weekdays and 1 weekend). Three multiple-pass 24-h dietary recalls.	Validity and Reproducibility	Differences in mean vitamin D and calcium between app and recall (paired-sample t-tests). Agreement between app and recall for vitamin D and calcium (Bland–Altman plot and Pearson correlation). Classification of mean intakes between app and recall (Wilcoxon signed-rank and intra-class correlations). Reproducibility of intakes estimated by the app over three time points (intra-class correlations, single measures, 2-way mixed and absolute agreement and Wilcoxon signed-rank tests).	Validity Correlations Vitamin D (IU/d); calcium (mg/g) agreement (app, recall); difference (app, recall) Food: (r = 0.84, *p* < 0.001; difference *p* = 0.20); (r = 0.63, *p* < 0.001; difference *p* = 0.49) Supplements: (r = 0.98, *p* < 0.001; difference *p* = 0.23); (r = 0.98, *p* < 0.001; difference *p* = 0.32) All sources: (r = 0.92, *p* < 0.001; difference *p* = 0.08); (r = 0.65, *p* < 0.001; difference *p* = 0.49) Intra-class correlations (ICC) Comparing binary classification ^1^ of 3-day mean recall vs. app ICC = 0.88 (95% CI: 0.80, 0.93; *p*< 0.001) (vitamin D) ICC = 0.50 (95% CI: 0.25, 0.68; *p* < 0.001) (calcium) Bland–Altman The percentage of points within LOA; 44% (vitamin D), 60% (calcium) Wilcoxon signed-rank tests Assessing quartile classification ^2^ of 3-day mean recall vs. app Z = –0.50, *p* = 0.62 (vitamin D); Z = –0.46, *p* = 0.65 (calcium) Reproducibility Intra-class correlations (ICC) Comparing app recording day 1 vs. 3 for mean intakes: ICC = 0.40 (95% CI: 0.14, 0.61; *p* = 0.002 (vitamin D) ICC = 0.22 (95% CI: –0.06, 0.47; *p* = 0.06 (calcium) Wilcoxon signed-rank tests Quartile mean intakes comparing days 1 vs. 2, 2 vs. 3 and 1 vs. 3 Vitamin D or calcium (*p* > 0.05 for all) Mean intakes for app recording day 1 vs. 3: Z = –1.19, *p* = 0.24 (vitamin D); Z = –1.76, *p* = 0.08 (calcium)
[28]	Mobile app A dietary app to self-monitor calcium consumption, to report the actual calcium levels in food and beverages that are typical of an Australian diet (5 days over 2-week period, 3 non-consecutive weekdays and 2 non-consecutive weekend days).	Usability	5-item usability questionnaire using 5-point Likert scales (strongly agree to strongly disagree).	Completed the usability questionnaire (*n* = 33, 83%). Easy and convenient to use (*n* = 20, 61%), app design intuitive and not confusing to use (*n* = 26, 79%), time-consuming (*n* = 14, 42%) Useful (*n* = 10, 30%)
[29]	Wearable device Three tri-axial accelerometers. Right Hip: GT1M, GT3X+ and GENEA. Left wrist: GT3X+. Right wrist: GENEA. GT1M 1 s epoch GT3X+ sampling rate 100 Hz. GENEAs sampling rate 80 Hz. Operating range 6 g. GRF 960 Hz using force plates.	Validity	Raw output between GT3X+ and GENA differed by activity and/or monitor (series of fully repeated measures ANOVAs—monitor × activity). Resultant peak g differed by hip or wrist across activities (ANOVAs (location × activity × monitor). Relationships between accelerometer output variables and force plate output variables (correlations, Fisher’s zr transformation).	Relationship between GRF ^3^ variables and GT1M counts: Peak impact force (r = 0.85, *p* < 0.05) Average resultant force (r = 0.73, *p* < 0.05) Peak loading rate (r = 0.76, *p* < 0.05). Relationship between GRF ^3^ variables and raw acceleration output: Hip vertical axis (GT3X+; GENEA) Peak impact force (r = 0.73, NS ^4^; r = 0.74, NS ^4^) Average resultant (r = 0.85, *p* < 0.05; r = 0.82, *p* < 0.05) Peak loading rate (r = 0.76, *p* < 0.05; r = 0.70, *p* < 0.05) Hip resultant (GT3X+; GENEA) Peak impact force (r = 0.73, NS ^4^; r = 0.73, NS ^4^) Average resultant (r = 0.87, *p* < 0.05; r = 0.85, *p* < 0.05) Peak loading rate (r = 0.70, *p* < 0.05; r = 0.63, *p* < 0.05) Wrist resultant (GT3X+; GENEA) Peak impact force GT3X+ (r = 0.59, NS ^4^); GENEA (r = 0.58, NS ^4^) Average resultant GT3X+ (r = 0.82, *p* < 0.05); GENEA (r = 0.87, *p* < 0.05) Peak loading rate GT3X+ (r = 0.79, *p* < 0.05); GENEA (r = 0.81, *p* < 0.05)
[30]	Wearable device Actigraph GT9X Link tri-axial accelerometers (right ankle, hip and wrist). Operating range ±8 g. Sampling rate 100 Hz. GRF 1000 Hz using force plates.	Validity	Accelerometer output and force plate output across wear sites (repeated measures correlations to assess concurrent validity).	Combined activities Hip Peak hip resultant acceleration and resultant loading rate (r = 0.74, *p* < 0.001, 95% CI: 0.718, 0.769) Peak hip resultant accelerations and resultant GRF ^3^ (r = 0.69, *p* < 0.001, 95% CI: 0.660, 0.720) Peak hip vertical accelerations and vertical GRF ^3^ (r = 0.50, *p* < 0.001, 95% CI: 0.455, 0.541) Peak hip vertical accelerations and loading rate (r = 0.57, *p* < 0.001, 95% CI: 0.525, 0.603) Wrist Peak wrist resultant accelerations and resultant loading rate (r = 0.17, *p* < 0.001, 95% CI: 0.113, 0.224) Peak wrist resultant acceleration and resultant GRF^3^ (r = 0.01, *p* = 0.815, 95% CI: −0.051, 0.064) Ankle Peak vertical acceleration and vertical GRF ^3^ (r = −0.09, *p* = 0.003, 95% CI: −0.145, −0.031) Peak vertical acceleration and vertical loading rate (r = 0.10, *p* = 0.001, 95% CI: 0.041, 0.155) Peak resultant acceleration and resultant GRF ^3^ (r = −0.09, *p* = 0.001, 95% CI: −0.151, −0.038) Peak resultant acceleration and resultant loading rate (r = 0.05, *p* = 0.063, 95% CI: −0.003, 0.111)

^1^ binary classification of 3-day mean, recall vs. app (vitamin D: ≤400 and ≥401 IU/d; calcium: ≤1000 and ≥1001 mg/d); ^2^ quartile classification of 3-day mean, recall vs. app (vitamin D: ≤200, 201–400, 401–600 and ≥601 IU/d; calcium: ≤800, 801–1000, 1001–2500 and ≥2501 mg/d); ^3^ ground reaction forces (GRF); ^4^ NS = not significant.

## Data Availability

Not applicable.

## Supplementary Materials

The following are available online at https://www.mdpi.com/article/10.3390/ijerph18115688/s1, Table S1: MEDLINE (Ovid). Search conducted January 2021. Table S2: List of excluded studies.
