# Supplementary material for: Validity of New Technologies That Measure Bone-Related Dietary and Physical Activity Risk Factors in Adolescents and Young Adults: A Scoping Review
Source: Int J Environ Res Public Health. 2021 May 26;18(11):5688. doi: 10.3390/ijerph18115688 (PMC8198820; doi:10.3390/ijerph18115688)
Supplement: Supplementary file 1 [file ijerph-18-05688-s001.zip › ijerph-1165511-supplementary.pdf]

Table S1. Search strategy in MEDLINE (Ovid), conducted in January 2021.

Search	Query	Records retrieved
#1 Population	Adolescent/ OR Students/ OR Young Adult/ OR Adolescen*.tw. OR Student*.tw. OR (Young* adj2 (Adult* OR Person* OR People* OR Wom?n OR M?n OR Female* OR Male*)).tw. OR Youth*.tw. OR Teen*.tw.	2898558
#2 Lifestyle (Diet and Physical Activity)	Life Style/ OR Adolescent Health/ OR Healthy Lifestyle/ OR Risk Factors/ OR exp Diet/ OR Calcium, Dietary/ OR Vitamin D/ OR exp Dairy Products/ OR Dietary Proteins/ OR Dietary Fiber/ OR Fruit/ OR Vegetables/ OR Caffeine/ OR Cola/ OR Tea/ OR Coffee/ OR Energy Drinks/ OR exp Exercise/ OR Resistance Training/ OR exp Sport/ OR (Lifestyle* OR Life Style*).tw. OR Risk Factor*.tw. OR Diet*.tw OR Nutrition*.tw. OR Calcium.tw. OR Vitamin D.tw. OR Dairy.tw. OR Milk.tw. OR Cheese*.tw. OR Yog?urt*.tw. OR Protein.tw. OR Fiber.tw. OR Fibre.tw. OR Fruit*.tw. OR Vegetable*.tw. OR Caffein*.tw. OR Cola.tw. OR Tea.tw. OR Coffee.tw. OR Energy Drink*.tw. OR Exercise*.tw. OR Resistance Training.tw. OR Sport*.tw. OR Physical Activit*.tw.	5535763
#3 Technologies	Wearable electronic devices/ OR Biomedical Technology/ OR Mobile Applications/ OR Smartphones/ OR Cell Phone/ OR Telemedicine/ OR Internet-Based Intervention/ OR Fitness Trackers/ OR exp Accelerometry/ OR Wearable*.tw. OR Sensor*.tw. OR eButton.tw. OR (Mobile* OR Smartphone*).tw. OR (App OR Apps* or Applications*).tw. OR Telemedicine.tw. OR (Activit* OR Fitness) adj2 (Monitor* OR Track* OR Device*).tw. OR Acceleromet*.tw. OR Pedometer*.tw. OR (Heart Rate Monitor* OR HR monitor*).tw. OR (Armband* OR Arm Band*).tw. OR Global Positioning Sensor*.tw. OR Ecological Momentary Assessment.tw. OR (GPS OR EMA).tw. OR MyFitnessPal.tw. OR MapMyRun.tw. OR Nike.tw. OR Fitbit.tw.	1070104
#4 Bone Health	Bone Density/ OR Bone Development/ OR exp Skeleton/ OR exp Bone Remodeling/ OR (Bone adj3 (Health or Densit* OR Mass OR Status OR Content OR Analysis OR Mineral OR Structur* OR Growth OR Model?ing OR Remode?ling OR Development OR Accrual OR Strength OR Architecture)).tw. OR Skeleton.tw. OR (PBM OR BMD).tw.	932139
#5	#1 AND #2 AND #3 AND #4	818
Limited to #Human and #English		753

Table S2. Excluded studies from the full-text screening.

No	Reference	Reason for exclusion
1	Weeks BK, Beck BR. The BPAQ: a bone-specific physical activity assessment instrument. *Osteoporos Int*. **2008**, *19*11, 1567-77.	Not study factor
2	Benito PJ, Neiva C, González-Quijano PS, Cupeiro R, Morencos E, Peinado AB, et al. Validation of the SenseWear armband in circuit resistance training with different loads. *Eur J Appl Physiol*. **2012**, *112*8, 3155-9.	Not study factor
3	Wundersitz DWT, Netto KJ, Aisbett B, Gastin PB. Validity of an upper-body-mounted accelerometer to measure peak vertical and resultant force during running and change-of-direction tasks. *Sports Biomech*. **2013**, *12*4, 403-12.	Not study factor
4	Pouliot-Laforte A, Veilleux LN, Rauch F, Lemay M. Validity of an accelerometer as a vertical ground reaction force measuring device in healthy children and adolescents and in children and adolescents with osteogenesis imperfecta type I. *J*. **2014**, *14*2, 155-61.	Not target group
5	Janz K, Letuchy E, Paulos R, Metcalf K, Burns T, Levy S. Comparison of accelerometer processing data for bone-related physical activity studies: Iowa bone development study. *Journal of Bone and Mineral Research Conference*. **2015**, *30*Supplement 1.	Full text not available
6	Meyer U, Ernst D, Schott S, Riera C, Hattendorf J, Romkes J, et al. Validation of two accelerometers to determine mechanical loading of physical activities in children. *J Sports Sci*. **2015**, *33*16, 1702-9.	Not target group
7	Park YJ, Lee SJ, Shin NM, Shin H, Yoon S, Jeon S, et al. Development of a smartphone application for promoting bone health in Korean young adult women: A pilot study. *Korean Journal of Adult Nursing*. **2015**, *27*4, 459-71.	Not in English
8	Setuain I, Martinikorena J, Gonzalez-Izal M, Martinez-Ramirez A, Gómez M, Alfaro-Adrián J, et al. Vertical jumping biomechanical evaluation through the use of an inertial sensor-based technology. *J Sports Sci*. **2016**, *34*9, 843-51.	Not study factor
9	Nedergaard NJ, Robinson MA, Eusterwiemann E, Drust B, Lisboa PJ, Vanrenterghem J. The Relationship Between Whole-Body External Loading and Body-Worn Accelerometry During Team-Sport Movements. *Int J Sports Physiol Perform*. **2017**, *12*1, 18-26.	Not study factor
10	Ziebart C, Giangregorio LM, Gibbs JC, Levine IC, Tung J, Laing AC. Measurement of peak impact loads differ between accelerometers - Effects of system operating range and sampling rate. *J Biomech*. **2017**, 58, 222-6	Not outcome factor
11	Raper DP, Witchalls J, Philips EJ, Knight E, Drew MK, Waddington G. Use of a tibial accelerometer to measure ground reaction force in running: A reliability and validity comparison with force plates. *J Sci Med Sport*. **2018**, *21*1, 84-8.	Not study factor

12	Toth LP, Park S, Springer CM, Feyerabend MD, Steeves JA, Bassett DR. Video-Recorded Validation of Wearable Step Counters under Free-living Conditions. *Med Sci Sports Exerc*. **2018**, *50*6, 1315-22.	Not study factor
13	Gomez-Carmona CD, Bastida-Castillo A, Gonzalez-Custodio A, Olcina G, Pino-Ortega J. Using an Inertial Device (WIMU PRO) to Quantify Neuromuscular Load in Running: Reliability, Convergent Validity, and Influence of Type of Surface and Device Location. *J Strength Cond Res*. **2020**, *34*2, 365-73.	Not study factor
14	Neugebauer JM, Collins KH, Hawkins DA. Ground Reaction Force Estimates from ActiGraph GT3X+Hip Accelerations. PLoS ONE. **2014**, 96, 8.	Not outcome factor
